# Multi-Omics Profiling in a Symptomatic Cohort Identifies Coordinated Biomarker Signatures in Ovarian Cancer Serum

**DOI:** 10.3390/diagnostics16142143

**Published:** 2026-07-08

**Authors:** Rachel Culp-Hill, Charles M. Nichols, Shannon Kilkenny, Mattie Goldberg, Enkhtuya Radnaa, Maria Wong, Moisés Zapata, Kian Behbakht, Benjamin G. Bitler, Anna Jeter, Vuna S. Fa, Kim Ekroos, Abigail McElhinny

**Affiliations:** 1 AOA Dx, Denver, CO 80221, USA; 2 Department of Obstetrics and Gynecology, Division of Reproductive Sciences, University of Colorado Anschutz Medical Campus, Denver, CO 80217, USA; 3 Lipidomics Consulting Ltd., 02230 Esbo, Finland

**Keywords:** ovarian cancer, multi-omics, lipidomics, metabolomics, biomarker discovery, early detection, symptomatic cohort, metabolic reprogramming, gangliosides, sphingolipids

## Abstract

**Background/Objectives:** Ovarian cancer (OC) is a leading cause of cancer-related mortality in women, largely driven by late-stage diagnosis. Five-year survival is just 30% for advanced-stage (III-IV) disease but exceeds 90% for early-stage disease, underscoring the critical need for effective early detection tools. Current standard-of-care biomarkers show limited sensitivity for early-stage OC and lack specificity in symptomatic populations. Most biomarker studies in OC serum evaluate single molecular classes or compare OC to healthy controls, limiting understanding of coordinated biological alterations in circulating proteins, lipids, and metabolites in clinically relevant populations. **Methods:** We performed integrated multi-omics profiling of serum from a retrospective, case–control cohort of women presenting with vague abdominal symptoms (VAS), including early- and late-stage OC, borderline tumors, benign gynecologic conditions including adnexal masses, GI disorders, and healthy controls. Protein biomarkers were quantified by ELISA, lipidomic profiling was performed by untargeted LC-MS, and ganglioside and metabolomic profiling were performed by semi-targeted LC-MS with metabolite annotation performed against a curated reference library. **Results:** Consistent with known limitations for early-stage OC detection, CA125 and HE4 levels overlapped substantially with benign gynecologic conditions. Additional proteins also showed limited separation in their expression between early-stage OC and symptomatic controls. In contrast, OC showed unique lipid and metabolite profiles: phospholipids and glycerolipids were decreased, and sphingolipid composition was altered. Borderline and benign conditions exhibited lipid profiles that fall between healthy and OC groups, suggesting a continuum of metabolic changes rather than distinct states between OC and non-OC controls. Sphingolipid alterations included changes in ceramides and sphingomyelins, along with broader dysregulation of ganglioside profiles, including an elevated GD2;O2-to-GD1;O2 ratio. Metabolic profiling showed decreased amino acids and enriched cysteine metabolism in OC, consistent with altered redox balance, along with changes in fatty acids and acyl-carnitines, suggesting altered lipid metabolism and inflammatory mechanisms. Lower levels of glycolytic and TCA cycle intermediates in OC suggested altered mitochondrial metabolism and energetic reprogramming. Pairwise comparisons revealed a gradient of significance between groups, with differences between OC and healthy controls across lipid classes (LPC, PC, PE, TG, SM), gangliosides (GD1, GD2, GD2/GD1 ratio), and metabolites (amino acids, Cys/CySS, TCA cycle); borderlines occupied an intermediate space. Integration of these datasets revealed coordinated cross-omics relationships, identifying links between metabolite, lipid, and protein features. Together, these connections highlight structured, system-level alterations related to lipid remodeling, redox balance, immune signaling, and energy metabolism that no single modality would have revealed in isolation. **Conclusions:** This study presents an integrated analysis of the lipidome, gangliosome, metabolome, and protein biomarkers within a single clinically relevant symptomatic cohort enriched with multiple stages and subtypes of OC. This multi-omics framework demonstrates that molecular alterations in OC are biologically interconnected across molecular classes. While these findings are discovery-based and require independent validation prior to clinical application, they support the development of clinically deployable multi-omics biomarker strategies for early detection and potential pathways for therapeutic intervention.

## 1. Introduction

Ovarian cancer (OC) remains one of the deadliest gynecologic malignancies and a persistent driver of female cancer mortality, ranking as the eighth leading cause of cancer-related deaths in women. Approximately 80% of patients are diagnosed only after metastatic spread has occurred [1]. Outcomes for advanced-stage disease are dismal, with 5-year survival rates below 30% [2] and relapse occurring in 75% of patients within 2 years [3]. Notably, over 80% of patients report vague abdominal symptoms (VAS) prior to diagnosis, but symptoms are non-specific and frequently overlap with benign conditions, resulting in an average diagnostic delay of ~9 months in the US [4].

The standard diagnostic workup relies heavily on serum biomarker testing, most commonly cancer antigen 125 (CA125). However, CA125 is elevated in only 50–62% of early-stage patients [5] and can be increased in benign and non-ovarian conditions [6], limiting its clinical utility. Despite decades of investigation, no blood-based diagnostic tool has achieved sufficient sensitivity and specificity for early-stage OC detection, and reliance on single biomarker approaches has failed to address this gap. An integrated multi-omics framework, which enables the simultaneous detection of complementary biological processes, offers a path to overcome the limitations of CA125 alone by exploiting metabolic alterations detectable through liquid biopsy.

Advances in high-resolution mass spectrometry (MS) now enable high-throughput detection of thousands of molecular features across large cohorts, supported by computational tools that can quickly interpret multi-omics datasets [7]. However, despite rapid growth in lipidomic and metabolomic studies, no metabolomics- or lipidomics-based cancer diagnostic assays have been translated to clinical practice. This disconnect highlights a critical gap between technological capability and clinical implementation in oncology. Given the heterogeneity of OC across histologic subtypes and stages, and that tumor-driven systemic metabolic changes are detectable in circulation, capturing multiple biomarker types is critical for identifying OC-specific signatures.

The “hallmarks of cancer,” originally described over 25 years ago [8], has evolved to position metabolic reprogramming as a central, intersecting driver of tumor biology, functioning not only as a consequence of tumor growth but as a driver of proliferation, invasion, and immune modulation [9,10]. Metabolites and lipids represent downstream functional consequences of these processes, but they also actively regulate these phenomena through energetic remodeling and lipid signaling [11]. Unlike upstream molecular layers such as the genome and transcriptome, the proteome and metabolome (including the lipidome) are more closely linked to the functional phenotype of a disease [12]. The shedding of proteins, metabolites, and lipids into circulation therefore allows for the detection of system-wide signatures associated with OC through blood-based multi-omics profiling.

Lipidomics, metabolomics, and proteomics have each been studied independently in OC serum. Protein biomarker research is extensive, with numerous studies and multiple blood-based clinical diagnostic assays aimed at improving CA125-based evaluation [13,14]. Parallel work has also identified lipidomic and metabolic alterations in OC [15], in pathways such as glycolysis and the tricarboxylic acid (TCA) cycle [16,17], amino acid metabolism [18], and fatty acid metabolism [19]. Gangliosides, lipids involved in membrane structure and cellular signaling, are also being investigated as circulating biomarkers of OC [20]. But despite this growing body of work, studies largely focus on single molecular classes in isolation. This fragmented approach highlights the need for integrated multi-omics strategies that offer a more complete metabolic picture. For example, while protein levels often reflect tumor-derived signaling, metabolomics reveals changes in energetic pathways and lipidomics informs changes in membrane composition. Recent studies show that combining multiple biomarker classes improves diagnostic performance compared to single-analyte approaches [21,22]. Integrated datasets within a single cohort are critical to identify interconnected molecular phenotypes that inform one another to build a more complete biological understanding of OC.

We hypothesized that integrating protein, lipidome, gangliosome, and metabolome profiling within a single cohort would reveal coordinated biological alterations not captured by single-omic analyses. In this study, we present an integrated multi-omics analysis within a clinically annotated cohort of women designed to represent patients with symptoms of OC. We integrate protein biomarkers (ELISA) with untargeted LC-MS profiling of the lipidome and semi-targeted LC-MS profiling of the gangliosome and metabolome. By capturing multiple layers of molecular biomarkers within the same cohort, this work provides a system-level view of serum-based analyte changes in OC and establishes a foundation for biologically informed multi-omics biomarker strategies for early detection.

## 2. Materials and Methods

### 2.1. Cohort Design

Vague abdominal (VAS) symptoms are common in both malignant and benign conditions, which can conflate ovarian cancer (OC) diagnoses. To characterize circulating biomarker alterations in women presenting with VAS, we designed a retrospective, case–control cohort of serum samples enriched for women with VAS and OC diagnoses across stages and subtypes. Serum specimens were obtained from multiple sources. OC cases included serous, endometrioid, clear cell, and mucinous subtypes; full histologic breakdown is provided in Supplementary Table S1. Borderline tumor samples (previously known as tumors of low malignant potential) were also included and are treated as a biologically intermediate group, distinguished from and analyzed in their own category distinctly from OC and the non-OC controls. Non-OC control samples were also included, encompassing gynecological conditions such as endometriosis, fibroids, cysts, and other benign adnexal masses. All OC and borderline cases were histologically confirmed. Throughout this manuscript, “non-OC controls” refers to healthy controls, benign gynecological conditions, and gastrointestinal (GI) disorders. Benign gynecologic conditions were confirmed at the time of surgical or clinical evaluation; the majority were pathologically confirmed, with a subset classified via imaging and clinical presentation. GI conditions including gastritis, colitis, and Crohn’s disease were also included, which can present with symptoms that overlap with OC. GI patients were previously diagnosed and were receiving treatment for their conditions at the time of collection. Age-representative, otherwise healthy female donors were also included to establish baseline metabolite and protein levels. Sample size was determined based on availability of clinically annotated specimens; no formal power calculation was performed, as this discovery-based study aimed to characterize biomarker profiles across disease stages. Cohort composition is summarized in Table 1. Clinical and demographic details are provided in Supplementary Table S1. Cohort samples were obtained from the University of Colorado Gynecologic Tissue and Fluid Bank (Institutional Review Board protocols #07-935 and 21-4787) and a commercial vendor. All participants gave written informed consent in accordance with the Declaration of Helsinki. All samples were de-identified prior to receipt and analysis. To mitigate sample quality variability from commercial vendors where possible, samples were excluded based on the following criteria: subjected to >2 freeze–thaw cycles, evidence of hemolysis or visible particulate matter, current cancer diagnosis other than ovarian cancer, previous ovarian cancer diagnosis, or pregnancy at the time of collection. Although commercial samples were obtained from female patients diagnosed with conditions described in Supplementary Table S2 and who presented with VAS aligning with the ovarian cancer symptom index, samples were not prospectively collected based on this index. No individuals were undergoing cancer-related treatment when blood was collected, and samples were collected prior to surgical intervention. For each sample, available data included cancer diagnosis, stage, and subtype (for OC cases), as well as age, sex, ethnicity, date of diagnosis, comorbidities, smoking history, and if available, medical history, reported symptoms, and symptom duration. Fasting status, menopausal status, and use of hormonal therapy were not available but are acknowledged as potential confounding variables for lipidomic and metabolomic profiles; these will be evaluated in future validation studies.

### 2.2. Lipidomics Sample Extraction

For lipidomic profiling, serum was extracted using 100% LC-MS-grade methanol (Thermo Fisher Scientific, Waltham, MA, USA). Methanol was spiked with the following internal standards: GD1b Ceramide (d18:1/18:0-d7), GD3 Ceramide (d18:1/18:0-d7), 15:0/18:1-d7 PE, 18:1-d7 LysoPE, and 18:1-d9 SM (Avanti Research, Alabaster, AL USA). Briefly, 20 μL serum was aliquoted into 2 mL microcentrifuge tubes and 180 μL of room-temperature (RT) spiked methanol was added. Samples were vortexed at maximum speed for 30 s and centrifuged at 18,000 rcf for 10 min at 4 °C. The resulting supernatant was transferred to glass autosampler vials. To generate a technical quality control mixture (Tmix) for global sample-level monitoring, 5 μL of supernatant was pooled from every 5th sample in randomized order. Samples were stored at −20 °C until analysis.

### 2.3. Lipidomics LC-MS

Reagents were purchased from Thermo Fisher Scientific unless otherwise specified. Analysis was performed by ultrahigh pressure liquid chromatography (UHPLC) MS on a Vanquish coupled to an Exploris 240 MS, using C18 reversed-phase chromatography and electrospray ionization (ESI). A 15 μL sample extract was injected onto a Kinetex 2.6 μm C18 LC Column (100Å, 100 × 2.1 mm, 2.7 μm) equipped with a SecurityGuard™ Ultracartridge (Phenomenex, Torrance, CA, USA) at 40 °C. Lipids were eluted using a 20 min gradient at 320 μL/min (40–90% B, A: 60:40 methanol/water + 10 mmol/L ammonium formate, B: 90:10 isopropanol/methanol + 10 mmol/L ammonium formate). For negative ion mode, three separate full scan MS experiments were run during the gradient: (1) 0–4 min: 180,000 resolution, 90–500 *m*/*z*; (2) 0–4 min: 120,000 resolution; 500–1700 *m*/*z*; and (3) 4–20 min: 120,000 resolution, 500–1700 *m*/*z*. Data-dependent MS/MS was performed using five dependent scans at 22,500 resolution with a scan range from 90 to 900 *m*/*z* (experiments 2 and 3). Higher-energy collisional dissociation (CD) was set to 20 with a 2 *m*/*z* isolation window. For positive ion mode, a full scan was performed at 120,000 resolution with a 300–1700 *m*/*z* scan range. Data-dependent MS/MS was performed using an 800 ms cycle time at 30,000 resolution and automatically detected scan range. Higher-energy collisional dissociation (CD, %) was set to 20 and 30 with a 1.5 *m*/*z* isolation window. EASY-IC was used for full scan acquisition in both ionization modes. Calibration was performed before analysis using Pierce FlexMix Calibration Solution. Tmix was injected every 10 samples to assess LC-MS stability and analytical precision. Internal standards maintained a coefficient of variation (CV) below 10% throughout the run.

### 2.4. Metabolomics Sample Extraction

For metabolomic profiling, serum was extracted using LC-MS-grade methanol:water (80:20 *v*/*v*, Thermo Fisher Scientific) spiked with the following internal standards: Metabolomics Amino Acid Mix, D-Glucose (U-^13^C_6_, 99%), sodium pyruvate (^13^C_3_, 99%), citric acid (1,5,6-carboxyl-^13^C_3_, 99%), α-Ketoglutaric acid, disodium salt (1,2,3,4-^13^C_4_, 99%), succinic acid (^13^C_4_, 99%), fumaric acid (^13^C_4_, 99%), L-malic acid (^13^C_4_, 99%) (Cambridge Isotope Laboratories) and palmitic acid–^13^C (1,2,3,4–^13^C_4_ labeled) (Cayman Chemical Company). Briefly, 20 µL serum was aliquoted into 2 mL Eppendorf tubes, and 480 µL of ice-cold extraction solvent was added. Samples were vortexed for 30 min and centrifuged at 12,000× *g* for 10 min at 4 °C. Supernatant was transferred to polypropylene autosampler vials. From every fifth sample, 5 μL of supernatant was removed in randomized order and added to a Tmix for global sample-level monitoring. Samples were stored at −20 °C until LC-MS analysis.

### 2.5. Metabolomics LC-MS

Reagents were purchased from Thermo Fisher Scientific unless otherwise specified. Analysis was performed by UHPLC-MS on a Vanquish coupled to an Exploris 240 MS, using C18 reversed-phase chromatography and ESI. A 10 μL sample extract was injected onto a Kinetex XB-C18 column (150 × 2.1 mm, 1.7 μm) equipped with a SecurityGuard™ Ultracartridge (Phenomenex, Torrance, CA, USA) at 45 °C. A 5 min gradient at 450 µL/min was used to elute metabolites (positive mode: 5–95% B, A: water/0.1% formic acid, B: acetonitrile/0.1% formic acid; negative mode: 0–100% B, A: 5% ACN, 95% water, 1 mM ammonium acetate, B: 95% ACN, 5% water, 1 mM ammonium acetate) For both ionization modes, full MS acquisition was performed at 70,000 resolution in the 65–975 *m*/*z* range, with 4 kV spray voltage, 45 sheath gas, and 15 auxiliary gas, operated in negative and then positive ion mode (separate runs). EASY-IC^TM^ was used for full scan acquisition in both ionization modes. Calibration was performed before analysis using Pierce FlexMix Calibration Solution (Thermo Fisher Scientific). Tmix was injected every 10 samples to assess LC-MS stability and analytical precision. Internal standards maintained a CV below 10% throughout the run.

### 2.6. LC-MS Data Analysis

Lipidomics data were processed using CompoundDiscoverer (v3.3 SP3, Thermo Fisher Scientific) for relative quantitation and lipid assignment against publicly available lipid databases. Databases used for putative library matches include LipidMaps, LipidBlast, Human Metabolome Database (HMDB), mzCloud, mzVault, MassList, and ChemSpider. To improve robustness and reduce technical artifacts, detected features were excluded based on the following filtering criteria: (i) lack of putative ID through library matching, (ii) present in less than 10% of samples within a cohort, (iii) exogenous origin, including drugs, synthetic compounds, dietary metabolites, and non-microbiome bacterial products, and (iv) high technical variability (CV > 50%) across technical quality controls. Feature annotation was performed using Compound Discoverer with database matching against publicly available spectral libraries. As with all untargeted annotations, these are putative identifications and may include mis-annotations or overreporting; therefore, feature verification using targeted methods will be required in future studies. Metabolomics data were converted from .raw to .mzML format using MSConvert [23]. Metabolite assignments, isotopologue distribution analysis, and correction for expected natural abundances of ^13^C isotopes were performed using MAVEN (Princeton, NJ, USA). Metabolite assignment was performed against an in-house standard library, as previously reported [24]. Annotation was limited to a predefined, curated database of 250 biologically relevant features containing features within metabolic pathways defined in the Kyoto Encyclopedia of Genes and Genomes (KEGG) [25].

### 2.7. Protein Immunoassays

Commercially available enzyme-linked immunosorbent assay (ELISA) kits for CA125, human epididymis protein 4 (HE4), folate receptor α (FOLR1), apolipoprotein A1 (ApoA1), and beta-2 microglobulin (B2M) were obtained from R&D Systems (Bio-Techne), and mucin 1 (MUC1) was measured using a kit from Thermo Fisher Scientific (Invitrogen). All assays were analytically verified according to manufacturer-recommended procedures. Immunoassays were performed on unextracted serum samples from each individual specimen. Samples were analyzed in technical replicates. Replicate measurements exhibiting >20% CV were repeated. Final concentrations were calculated according to manufacturer-provided standard curves.

### 2.8. Statistical Analysis

Data visualization and statistical analysis were performed using GraphPad Prism (GraphPad v10.4) and Metaboanalyst [26]. Outlier analysis was performed in GraphPad Prism using robust regression and outlier removal (ROUT, Q = 1%) and applied across all groups simultaneously. Identified outliers were removed from graphical visualization to improve interpretability of low-abundance species but were retained in statistical analyses. Scatter plot comparisons were performed using the Kruskal–Wallis test, with Dunn’s multiple comparisons post hoc analysis where applicable. Statistical significance was defined as *p* ≤ 0.05 (*), *p* ≤ 0.01 (**), *p* ≤ 0.001 (***), and *p* ≤ 0.0001 (****).

### 2.9. Multi-Omics Correlation Network Analysis

Matched metabolomics, lipidomics, and protein data were integrated to construct a multi-omics correlation network. Lipid features were restricted to those with confirmed MS/MS identification to improve annotation confidence. A precomputed correlation matrix containing all molecular features was analyzed in R. Pairwise feature correlations were converted into an edge list, retaining only unique feature pairs and excluding self-correlations. Edges were filtered using an absolute correlation threshold (|r| ≥ 0.55) to focus on moderate-to-strong associations. The resulting network was visualized in Cytoscape (v3.10.4) with node color indicating data type and edge width and color representing correlation magnitude and direction. A Prefuse force-directed layout was applied, followed by manual adjustment to improve visual clarity.

## 3. Results

### 3.1. Protein Biomarker Levels

Six protein biomarkers were quantified using commercial immunoassay kits: ApoA1, B2M, CA125, FOLR1, HE4, and MUC1. CA125 and HE4 are currently incorporated into clinical risk assessment for OC [27,28], while ApoA1 [29], B2M [30], FOLR1 [31], and MUC1 [32] have been investigated as promising diagnostic or therapeutic biomarkers in OC.

**Figure 1:**
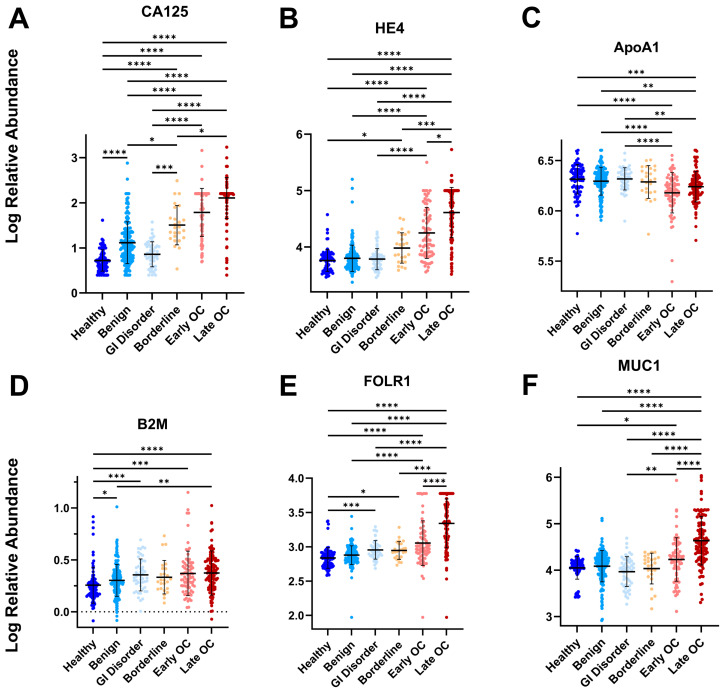
ELISA-based protein biomarker levels. Signatures of serum proteins compared across 6 groups: Healthy controls (healthy, dark blue), benign gynecological disorder (benign, medium blue), GI disorder (GI, light blue), borderline tumors (borderline, yellow), early OC (early-stage ovarian cancer, pink), late OC (late-stage ovarian cancer, red). Immunoassay-based protein biomarkers were quantified and then log10-transformed and visualized by scatter plot. (**A**) CA125. (**B**) HE4. (**C**) ApoA1. (**D**) B2M. (**E**) FOLR1. (**F**) MUC1. Error bars indicate mean and SD. Statistical significance assessed using Kruskal–Wallis and Dunn’s multiple comparisons test, with *p*-values indicating pair-wise group differences. * *p* ≤ 0.05, ** *p* ≤ 0.01, *** *p* ≤ 0.001, **** *p* ≤ 0.0001.

Consistent with prior reports, CA125 and HE4 demonstrated the largest increases in OC relative to non-OC groups (healthy controls, benign gynecological conditions, and GI disorders) as well as borderline tumors with CA125 elevated 15.3-fold in early-stage and 31.8-fold in late-stage OC relative to healthy controls, and HE4 elevated 2.2-fold and 9.0-fold, respectively (Figure 1A,B). However, elevations in CA125 were also observed in benign gynecologic conditions, reflecting the known overlap between malignant and non-malignant adnexal masses that confounds OC diagnosis. Levels of HE4 in early-stage OC also overlap with benign gynecologic conditions and borderline tumors. This overlap for both CA125 and HE4 underscores the limitations of relying on single-protein biomarkers for definitive discrimination, particularly in symptomatic populations.

Among the other evaluated proteins, ApoA1 showed subtle but distinct decreases in OC, with levels reduced 0.72-fold in early-stage OC and 0.80-fold in late-stage OC relative to healthy controls (Figure 1C). Minimal differences were found between the healthy, benign, GI, and borderline groups. FOLR1 and MUC1 were both elevated in late-stage OC (3.3-fold and 3.1-fold relative to healthy controls, respectively), with more modest elevation in early-stage disease (1.4-fold and 1.3-fold, respectively). FOLR1 and MUC1 also showed minimal differences between healthy, benign, GI, and borderline groups (Figure 1E,F). B2M, while statistically significant, showed the least difference between all groups. Early- and late-stage OC were just 1.3-fold and 1.4-fold increased relative to healthy controls, respectively, with substantial overlap between all groups (Figure 1D).

Together, these findings reinforce the known challenges inherent to single-analyte biomarkers for the detection of OC. While established markers CA125 and HE4 show stage-associated increases, substantial overlap of their levels limits their utility in symptomatic, benign conditions. These observations support expanding beyond protein-only approaches by incorporating novel biomarker classes such as lipids, gangliosides, and small-molecule metabolites to better characterize the systemic phenotype of OC in comparison to symptomatic individuals.

### 3.2. Lipidomic Profiling

To characterize the lipidome of OC serum, we performed untargeted high-resolution UHPLC-MS lipidomic profiling using data-dependent MS/MS to maximize lipid coverage across the cohort.

**Figure 2:**
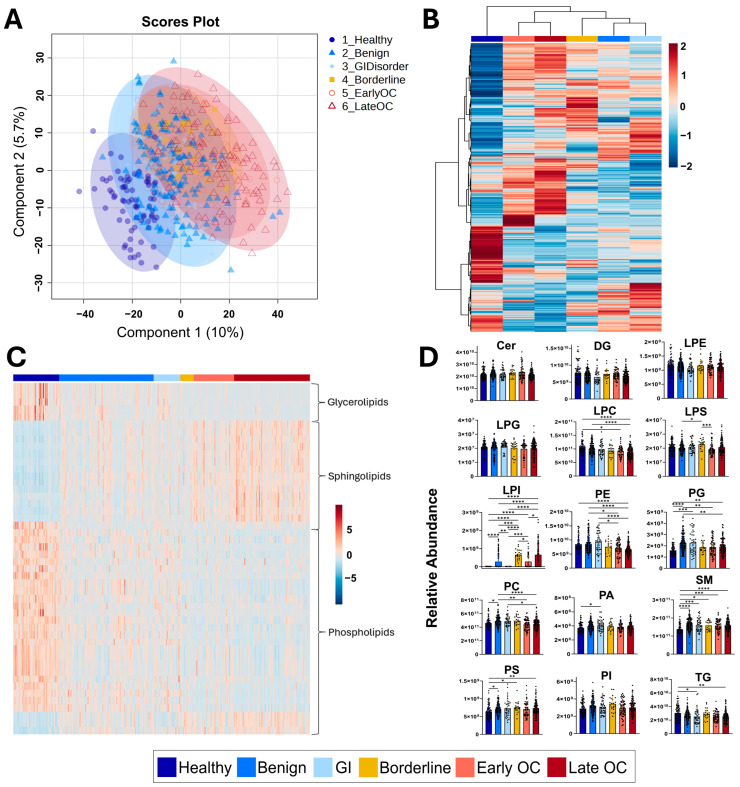
Lipidomic profiling of OC serum. Signatures of serum lipids compared across 6 groups: healthy controls (healthy, dark blue ●), benign gynecological disorder (benign, medium blue ▲), GI disorder (GI, light blue ♦), borderline tumors (borderline, yellow ■), early OC (early-stage ovarian cancer, pink ○), late OC (late-stage ovarian cancer, red Δ). (**A**) PLS-DA of all groups. (**B**) Heatmap of average lipid features across all 6 groups. Features were median-normalized and auto-scaled (z-score normalization). Hierarchical clustering for both averaged groups and features were performed using Euclidean distance and Ward’s method. (**C**) Heatmap of top 50 ANOVA-selected features across all 6 groups by lipid class. Features were median-normalized and auto-scaled (z-score normalization). Hierarchical clustering performed on features using Euclidean distance and Ward’s method. (**D**) Scatter plots depicting relative abundance of summed lipid classes. Error bars indicate mean and SD. Statistical significance assessed using Kruskal–Wallis and Dunn’s multiple comparisons test, with *p*-values indicating pair-wise group differences. Lipids were putatively identified using database matching in Compound Discoverer; additional studies are required to verify their identifications. These assignments should be interpreted with caution and require confirmation using targeted analytical approaches. * *p* ≤ 0.05, ** *p* ≤ 0.01, *** *p* ≤ 0.001, **** *p* ≤ 0.0001.

Partial least squares discriminant analysis (PLS-DA) demonstrated unique patterns of separation across the clinical groups (Figure 2A). Healthy controls clustered separately from borderline tumors, early-stage OC, and late-stage OC, with partial overlap observed with benign gynecological conditions and GI disorders. Interestingly, borderline tumors, early-stage OC, and late-stage OC cluster closely, suggesting shared lipidomic alterations.

To understand the scale of lipidomic alterations across the groups, we generated a heatmap of all detected lipid features using group-averaged intensities with hierarchical clustering (Figure 2B). Consistent with multivariate analysis, healthy controls displayed a lipid profile unique from both symptomatic and malignant groups. Furthermore, early-stage and late-stage OC profiles clustered together, and malignant groups separated clearly from borderline tumors, benign gynecological conditions, and GI disorders.

We also examined the top 50 lipid features by ANOVA, without hierarchical clustering of clinical groups (Figure 2C). Among the top 50 lipid features, 76% were decreased in OC relative to healthy controls, consistent with the conclusions of previous reports describing global lipid depletion in OC serum [21]. We also observed a unique profile for borderline tumors, benign gynecological conditions, and GI disorders, marking a “middle ground” between healthy controls and OC. This phenomenon reflects biological overlap between malignant and non-malignant serum profiles, supporting a disease-specific continuum that may be informative for risk stratification in symptomatic populations, rather than strict binary classification.

To assess class-level lipidomic alterations, lipid species were aggregated by class and compared across all groups (Figure 2D). We observed significant changes across multiple lipid classes: sphingomyelins (SM) increased across all groups relative to healthy controls, with increases of ~1.2-fold early-stage and ~1.15-fold in late-stage OC. Triglyceride (TG), lysophosphatidylcholine (LPC), and phosphatidylethanolamine (PE) were decreased in OC, with the largest reductions in late-stage disease (0.81-fold relative to healthy controls.) Phosphatidylcholine (PC) was decreased relative to benign and GI groups (0.93-fold in both early- and late-stage OC vs. benign), also demonstrating that PC is increased in benign, GI, and borderline groups relative to healthy controls. Lysophosphatidylinositol (LPI) showed a distinct profile for benign gynecological conditions, borderline tumors, and OC relative to healthy controls and GI disorders, suggesting that this subclass may reflect the growth of any adnexal mass, regardless of malignancy.

Together, these analyses demonstrate clear lipidomic remodeling in OC serum alongside overlap with non-malignant clinical groups. Given the observed increase within the sphingomyelin subclass and our previous work which reports increased levels of disialo-gangliosides in OC serum by ELISA [20], we directed our analysis toward ganglioside-specific alterations, also known as the gangliosome.

### 3.3. Gangliosome Profiling

Gangliosides, sialic-acid containing glycosphingolipids, are a unique subclass of sphingolipids that are primarily associated with neural tissue but also present in circulation. Recent studies have implicated gangliosides in OC biology, as they are critical to cellular signaling and can be shed from the tumor into circulation [33]. While lipidomics studies in OC serum are increasingly common, alterations in the gangliosome have not been well characterized in this context. We therefore characterized gangliosides across all clinical groups to determine whether changes in ganglioside composition contribute to the sphingolipid alterations we observed and evaluate their relevance to OC biology.

**Figure 3:**
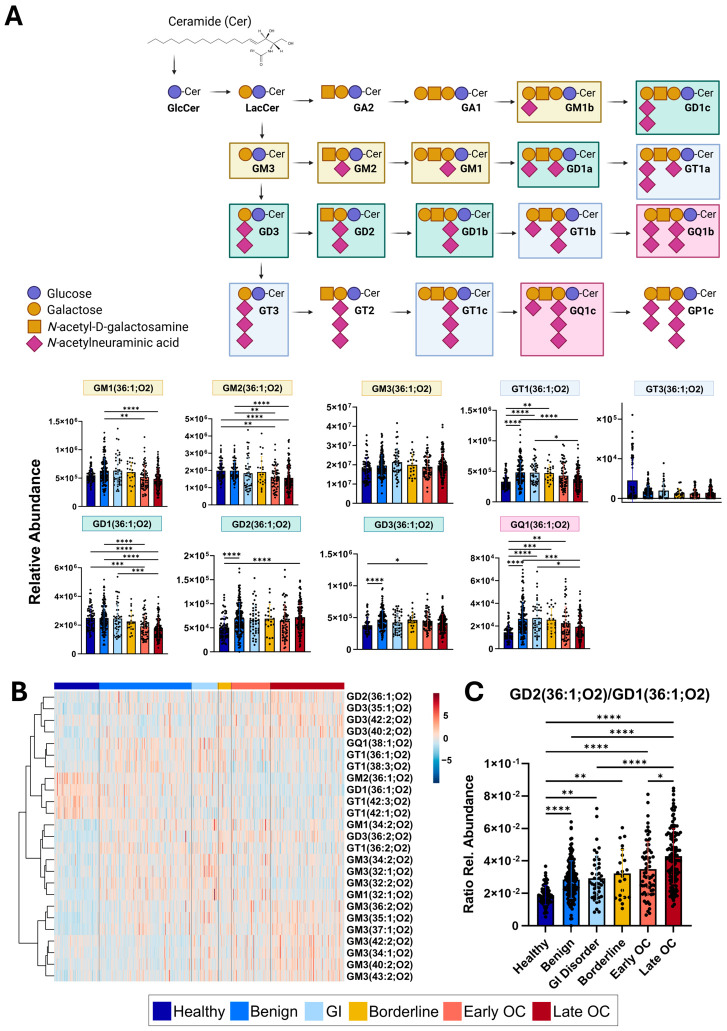
Alterations in the gangliosome of OC serum. Signatures of serum gangliosides compared across 6 groups: healthy controls (healthy, dark blue), benign gynecological disorder (benign, medium blue), GI disorder (GI, light blue), borderline tumors (borderline, yellow), early OC (early-stage ovarian cancer, pink), late OC (late-stage ovarian cancer, red). (**A**) Scatter plots depicting alterations across all 6 groups for intermediates of the ganglioside metabolism pathway. GMs are highlighted in yellow, GDs in green, GTs in blue, and GQ in pink. (**B**) Heatmap of top 25 ANOVA-selected gangliosides across all 6 groups. Features were median-normalized and auto-scaled (z-score normalization). Hierarchical clustering on features was performed using Euclidean distance and Ward’s method. (**C**) Scatter plot depicting the ratio of disialo-gangliosides GD2 (36:1;O2) to GD1 (36:1;O2), which may suggest altered activity of the enzyme B3GALT4, which converts GD2 to GD1, as shown in (**A**). Error bars indicate mean and SD. Statistical significance assessed using Kruskal–Wallis and Dunn’s multiple comparisons test, with *p*-values indicating pair-wise group differences. * *p* ≤ 0.05, ** *p* ≤ 0.01, *** *p* ≤ 0.001, **** *p* ≤ 0.0001.

Figure 3A depicts the biosynthesis of gangliosides, illustrating the sequential conversion of ceramides to complex glycosphingolipids and gangliosides. To avoid the confounding effects of summing all species within a subclass, adjacent scatter plots display corresponding levels of representative ganglioside species with a d36:1;O2 sum composition, the most abundant molecular form in this study. GD1 was decreased in early- and late-stage OC relative to healthy controls (0.79-fold and 0.70-fold, respectively), while GD2 was modestly elevated (1.40-fold and 1.52-fold). We also observed decreases in GM1 and GM2 for early- and late-stage OC relative to non-OC. Other species such as GD3, GT1, and GQ1 show clear differences between healthy controls and all other groups but more modest separation between malignant and benign conditions.

We then generated a heatmap of the top 25 gangliosides by ANOVA *p*-value to highlight the gangliosides that show the largest differences across groups. Variation in the gangliosome can be clearly observed, and while most gangliosides are elevated in early- and late-stage OC, 4 of the 25 gangliosides were markedly decreased. One of these species is GD1(36:1;O2), which can be produced via conversion of GD2 by the glycosyltransferase B3GALT4 [34]. Additionally, as GD2(36:1;O2) was increased in OC relative to non-OC controls, we evaluated whether the GD2(36:1;O2)-to-GD1(36:1;O2) ratio would be consistent with our hypothesis of decreased function of B3GALT4 (Figure 3C). The GD2/GD1 ratio was elevated 1.63-fold in early-stage and 2.22-fold in late-stage OC relative to healthy controls, with borderline tumors showing an intermediate elevation of 1.68-fold, consistent with the continuum observed across other molecular classes. An increase in this ratio for individuals experiencing symptoms of OC or individuals diagnosed with OC may suggest altered enzymatic function. While there is limited data surrounding B3GALT4 in OC, it has been implicated in other cancer types and is associated with tumor microenvironment remodeling through lipid raft [35]. However, shifts in the tumor microenvironment to enable cancer cell growth and survival are also reflected in the metabolome and can directly influence the lipidome. Given that lipid remodeling and metabolism are deeply interconnected, we next explored the metabolome of OC serum.

### 3.4. Metabolomics Profiling

Aberrant metabolic regulation is recognized as a hallmark of cancer [36], as oncogenes and tumor suppressors reprogram metabolic function, influencing proliferation, survival, and differentiation [37]. We therefore utilized semi-targeted metabolomics to characterize metabolic alterations within this cohort.

**Figure 4:**
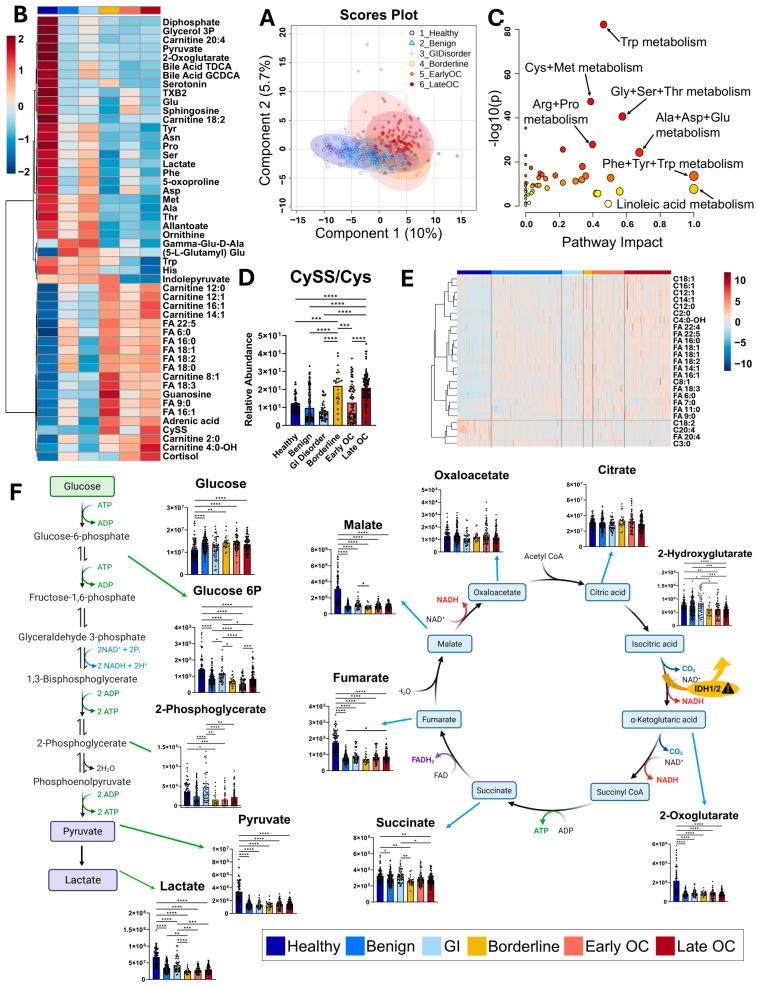
Metabolic profiling of OC serum. Metabolic signatures compared across 6 groups: healthy controls (healthy, dark blue ●), benign gynecological disorder (benign, medium blue ▲), GI disorder (GI, light blue ♦), borderline tumors (borderline, yellow ■), early OC (early-stage ovarian cancer, pink ○), late OC (late-stage ovarian cancer, red Δ). (**A**) PLS-DA analysis of all groups. (**B**) Heatmap of group averaged top 50 ANOVA-selected features across all 6 groups. Features were median-normalized and auto-scaled (z-score normalization). Hierarchical clustering on features was performed using Euclidean distance and Ward’s method. (**C**) Metabolic pathway analysis of serum performed using KEGG-based enrichment and topology analysis. Circle color indicates significance (-log10 *p*-value), and circle size represents pathway impact. (**D**) Scatter plot of the ratio of cystine (CySS) to cysteine (Cys) across all 6 groups. (**E**) Heatmap of top 50 fatty acids and acyl-carnitines by ANOVA across all 6 groups. Hierarchical clustering performed using Euclidean distance and Ward’s method. (**F**) Scatter plots depicting alterations across all 6 groups for glycolytic intermediates (left) and TCA cycle intermediates (right). Error bars indicate mean and SD. Statistical significance assessed using Kruskal–Wallis and Dunn’s multiple comparisons test, with *p*-values indicating pair-wise group differences. * *p* ≤ 0.05, ** *p* ≤ 0.01, *** *p* ≤ 0.001, **** *p* ≤ 0.0001.

PLS-DA showed group-level clustering across the clinical groups, but patterns were more subtle than those observed in the lipidomics data (Figure 4A). Healthy controls separated from all other groups but overlapped with benign gynecological conditions. GI, borderline, early-stage OC, and late-stage OC clustered closely, suggestive of metabolic profiles distinct from healthy and benign groups.

To assess the scale of metabolic alterations across groups, we generated a heatmap using group-averaged intensities with hierarchical clustering (Figure 4B). Consistent with trends observed in the PLS-DA, healthy controls displayed a metabolic profile unique from all other groups. Early-stage OC, late-stage OC, and borderline tumors displayed similar profiles, distinct from benign and GI groups.

Pathway analysis comparing OC to non-OC controls revealed enrichment in linoleic acid and amino acid metabolism pathways (Figure 4C). Supplementary Figure S1 displays scatter plots of all detected amino acids. Amino acid levels were largely decreased in early- and late-stage OC, whereas healthy controls exhibited the highest levels, and benign, GI, and borderline groups showed intermediate levels. Notably, cysteine was an exception, showing no significant differences between groups, while cystine was highly variable. As cysteine and methionine metabolism pathways were among the most highly impacted, this reflects altered redox balance. We therefore analyzed the ratio of cystine to cysteine (CySS/Cys) and observed that the ratio was elevated 1.98-fold in late-stage OC relative to healthy controls, with borderline tumors showing the highest ratio overall (2.30-fold vs. healthy.) Furthermore, borderline and OC groups displayed elevated ratios relative to healthy, benign, and GI groups (Figure 4D), suggesting redox metabolism may be involved in borderline and OC tumor biology.

As linoleic acid metabolism was also enriched, we generated a heatmap of the top 25 fatty acids and acyl-carnitines by ANOVA to visualize alterations in fatty acid and carnitine metabolism (Figure 4E). Overall, benign, borderline, and OC groups displayed elevated fatty acids and carnitines relative to healthy controls and GI disorders. However, a few species were decreased in all non-healthy groups: arachidonic acid (FA 20:4), arachidonyl-carnitine (C20:4), linoleic acid (FA 18:2), propionyl-carnitine (C3:0). These findings are consistent with reports that describe elevated fatty acid metabolism in cancer serum [38] and suggest that those elevations extend to borderline tumors and benign gynecological conditions. Additionally, decreased arachidonic acid and arachidonyl-carnitine across all non-healthy groups may indicate changes in arachidonic acid metabolism, which has been extensively studied in cancer biology, including OC [39].

Finally, to further evaluate energy metabolism within these groups, we examined glycolytic and TCA cycle intermediates (Figure 4F). Downstream glycolytic intermediates were markedly decreased in OC relative to healthy controls: glucose-6-phosphate (0.34-fold in early-stage, 0.53-fold in late-stage), pyruvate (0.41-fold in both stages), and lactate (0.39-fold in early-stage, 0.40-fold in late-stage), despite glucose being slightly elevated (1.41-fold in early-stage, 1.32-fold in late-stage). TCA cycle intermediates showed similar patterns, with α-ketoglutarate (0.41-fold in early-stage, 0.39-fold in late-stage), fumarate (0.48-fold in early-stage, 0.51-fold in late-stage), and malate (0.33-fold in early-stage, 0.32-fold in late-stage) all substantially decreased. However, glucose was slightly elevated across all groups relative to healthy controls, while downstream intermediates (glucose-6-phosphate, 2-phosphoglycerate, pyruvate, and lactate) were decreased. These patterns suggest that all non-healthy groups (benign, GI, borderline, early OC, and late OC) downregulate glycolysis. We also observed TCA cycle alterations: while citrate, succinate, and oxaloacetate remained relatively unchanged, α-ketoglutarate, fumarate, and malate were notably decreased in benign, GI, borderline, early-stage OC, and late-stage OC, which is consistent with altered mitochondrial metabolism.

Together, these metabolomic and lipidomic patterns indicate altered energy metabolism, redox balance, and membrane composition, reflecting broad metabolic reprogramming across both malignant and non-malignant conditions. These findings highlight the value of integrating multiple molecular classes to better characterize disease-associated biological processes in OC.

### 3.5. Multi-Omics Integration

**Figure 5:**
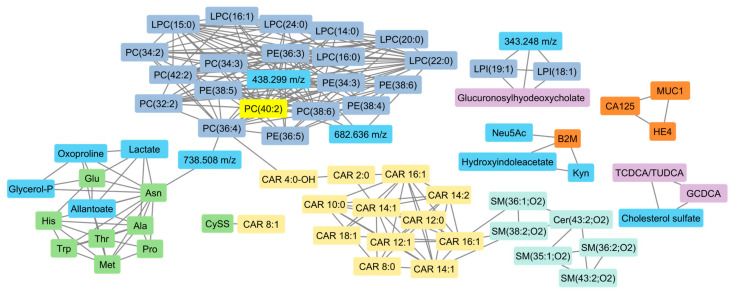
Multi-omics correlation network integrating metabolomic, lipidomic, and protein features. A correlation-based network was constructed from matched metabolomics, lipidomics (MS2-confirmed features only), and protein data using an absolute correlation threshold of |r| ≥ 0.55. Nodes represent individual molecular features and are colored by class: amino acids (green), bile acids (purple), acyl-carnitines (yellow), proteins (orange), sphingolipids (light green), phospholipids (light purple), and all other features (blue). Edges indicate pairwise correlations, with thickness proportional to correlation strength.

To examine cross-omics relationships, a correlation network was constructed integrating matched metabolomic, lipidomic, and protein data (Figure 5). Only lipid features with MS/MS data from Compound Discoverer were integrated. Using a threshold of |r| ≥ 0.55, we observed extensive cross-omics associations across molecular classes. Protein markers formed discrete clusters, including CA125, HE4, and MUC1, as well as a separate cluster linking B2M with N-acetylneuraminate and two tryptophan pathway metabolites (hydroxyindoleacetate and kynurenine). Lipid and metabolite interactions were prominent: phospholipid species (PE, PC, LPC) were highly interconnected, and sphingolipids formed a distinct cluster with connections to long-chain acyl-carnitines (C14:1, C16:1). Carnitines also formed a cluster spanning short- to long-chain species, with connections to phospholipids. Additionally, amino acids formed a cluster that also included lactate, oxoproline, glycerol phosphate, allantoate, and diphosphate. Notably, this amino acid cluster links to the phospholipid cluster via an unknown feature (738.508 *m*/*z*). Bile acids also formed distinct clusters, grouping with cholesterol sulfate, consistent with their shared biochemical origin. A separate cluster comprised lysophosphatidylinositols, a bile acid, and an unidentified feature. Finally, we observed a connection between cystine and C8:1 carnitine.

## 4. Discussion

High mortality rates for OC are driven by late-stage diagnoses. Early detection has been shown to reduce cancer-related mortality, but the current lack of effective diagnostic tools is worsening patient outcomes. While tumor markers like CA125 and HE4 remain central to clinical practice [5,40,41], they show limited sensitivity for early-stage disease. Accurate diagnosis is further complicated by tumor heterogeneity, which limits the utility of any single biomarker [42]. CA125 is also elevated in benign gynecological conditions such as benign adnexal masses, acute pelvic inflammation, and menstruation [6], resulting in poor specificity for symptomatic populations. While HE4 improves performance in diagnostic algorithms like ROMA, early-stage sensitivity remains insufficient [43]. Even multivariate protein panels like OVA1 and Overa have not overcome these limitations [44]. Therefore, novel diagnostic tools must leverage the power of multi-omics to move past single biomarker strategies and improve early-stage detection.

The proteins evaluated in this study were selected based on their potential utility in early OC detection [5,6,45,46,47,48,49], aligning with key processes involved in tumor initiation and disease progression. Inflammation plays a well-established role in cancer biology, with epidemiological estimates attributing 15–25% of cancer-related deaths to chronic inflammation or infectious disease [50,51,52]. Several of the proteins measured in this study have been directly linked to inflammation in OC: CA125 influences neutrophil activity and amplifies the immune response, MUC16 is upregulated by inflammatory stimuli through NF-κB activation, and HE4 is regulated by NF-κB through inflammatory cytokines like TNF-α [53]. Our findings are consistent with previously published work and reinforce the limitations of protein-only strategies. CA125 and HE4 showed increases in OC and borderline tumors but overlapped with benign gynecological conditions, limiting their utility for early-stage detection. ApoA1 was decreased in OC, consistent with previous work linking reduced ApoA1 to altered lipid transport, inflammation, and oxidative stress [5,6,45,49]. FOLR1 and MUC1 were elevated in late-stage OC, which aligns with their established roles in folate uptake [6,54,55], proliferative signaling, and epithelial remodeling [41,56], but they showed substantial overlap across early-stage OC and non-OC controls. B2M showed modest differences across groups, consistent with its role in immune activation, which may be increased in non-healthy groups. Taken together, while these findings reflect known changes in tumor signaling and inflammatory processes in OC, even an expanded protein panel has clear limitations for early-stage detection.

LC-MS-based lipidomic profiling has been sparse, with most studies comparing OC cases to healthy controls in small, clinically homogenous cohorts. These studies have identified altered phospholipid and sphingolipid remodeling in OC serum [57,58], but their application to real-world clinical settings is limited, as they do not include symptomatic patients undergoing evaluation for OC. Our findings are broadly consistent with these prior studies: OC showed decreased phospholipids and glycerolipids relative to non-OC controls, along with increased class-specific sphingolipid alterations, suggesting that previously reported lipid remodeling is reproducible across platforms and cohort designs. However, the inclusion of benign gynecologic conditions and borderline tumors reveals an important but understudied continuum of lipid profiles based on distinct disease states. The lipidomic signatures of these groups occupy an intermediate space between healthy controls and OC, with borderline tumors clustering closely with OC. This suggests that lipid remodeling reflects systemic changes associated with adnexal mass biology and captures a continuum from healthy to benign to malignant states. These data support the utility of at least some of these features for risk stratification. Further molecular dissection using targeted LC-MS is underway to evaluate the utility of high-performing lipids for distinguishing cancer from non-cancerous conditions in a clinical diagnostic assay.

In comparison to the relatively few studies that have evaluated the broader lipidome in OC serum, far fewer have examined lipid subclasses such as gangliosides. Gangliosides are involved in membrane biology and signaling, and species such as GD2 and GD3 have been implicated in tumor progression across multiple cancer types. Prior work has described elevated GD2 and GD3 in OC tumor tissue and circulation, suggesting that ganglioside biosynthesis is dysregulated in OC [20]. However, these analyses have focused on individual species rather than the broader “gangliosome”. In the present study, we observed altered ganglioside pathway activity in OC relative to non-OC controls, including increased GD2(36:1;O2) and decreased GD1(36:1;O2). The elevated ratio of GD2 to GD1 suggests decreased activity of glycosyltransferases such as B3GALT4, which converts GD2 to GD1. While serum data cannot provide a direct measure of enzymatic function, this profile, alongside changes in other ganglioside species, is consistent with alterations within the ganglioside biosynthetic pathway. However, we again observed overlap between early-stage OC, non-OC controls, and borderline tumors, emphasizing that a single biomarker class is not sufficient to distinguish early-stage OC from symptomatic individuals.

Metabolic reprogramming is a hallmark of cancer [9], and metabolic changes have been reported in OC serum [16,17,18,19]. However, in line with previous lipidomic studies, these analyses have often compared OC serum to healthy controls and focused on a limited panel of metabolites. Even fewer studies integrate lipidomics and metabolomics across the same cohort. In the present study, we observed that circulating amino acids were decreased in OC, which is consistent with previous work and suggests amino acids could be depleted for the purposes of nucleotide and protein synthesis. One such depleted amino acid was tryptophan, which is metabolized to kynurenine. Kynurenine has been shown to drive immune suppression in high-grade serous OC (HGSOC) through aberrant expression of indoleamine 2,3-dioxygenase 1 (IDO1) and tryptophan 2,3-dioxygenase (TDO2) [59]. Additionally, pathway analysis specifically highlighted cysteine and methionine metabolism, and together with an elevated ratio of cystine/cysteine in borderline tumors and OC groups, this suggests that redox balance is altered. Fatty acid and acyl-carnitine levels were also increased in benign gynecological conditions, borderline tumors, and OC, particularly linoleic acid and palmitic acid, which may support energetic demands and increased lipid production. Additionally, decreased arachidonic acid and arachidonyl-carnitine in all symptomatic groups is indicative of altered eicosanoid metabolism, which is critical to inflammatory processes and aligns with the changes in inflammation-associated proteins discussed above. Decreased levels of downstream glycolytic intermediates and altered TCA cycle function also suggest that OC may utilize metabolic compensation beyond aerobic glycolysis. Notably, previous studies have identified that carnitine palmitoyl transferase 1 (CPT1)—a mitochondrial enzyme involved in fatty acid oxidation—is elevated in HGSOC, which may be reflected in our observed alterations in both fatty acid metabolism and TCA cycle intermediates [60]. These findings link alterations in amino acid metabolism, lipid remodeling, and inflammatory signaling, suggesting that coordinated metabolic reprogramming spans multiple molecular classes. Metabolic dysfunction is an underexploited hallmark of OC biology, and the observation of multiple biomarker classes simultaneously offers a comprehensive view of metabolic changes—for example, alterations in fatty acid metabolism can inform changes in the lipidome and underscore the biological implications of inflammation-associated proteins. These findings therefore show that multi-omics integration can provide context for a biologically informed diagnostic strategy. In fact, previous studies have already reported diagnostic performance, including high AUCS alongside improved sensitivity and specificity relative to CA125 and HE4, demonstrating that combining proteins with lipids using a machine learning model results in dramatic improvement in the detection of early-stage OC in a symptomatic population [21,22]; the current work focuses on the biological characterization that underlies and informs those diagnostic strategies.

The correlation network (Figure 5) reveals extensive cross-omics connections, highlighting relationships between metabolites, lipids, and proteins. Several associations corroborate known alterations in OC biology while revealing links between processes that have typically been studied in isolation. Notably, B2M clustered with metabolites involved in tryptophan metabolism, consistent with the role of tryptophan metabolism in immune regulation and OC biology [59]. Amino acid metabolism was also identified as a central hub, with connections to glycolytic intermediates such as lactate, glycerol phosphate, and diphosphate, supporting a link between energy metabolism and biosynthesis. Connections between the lipidome and metabolome were also identified: phospholipids, carnitines, and sphingolipids formed individual clusters but were also interconnected, suggesting coordination between membrane remodeling, fatty acid transport, and mitochondrial metabolism. Additional clusters reflected known biochemical relationships, including bile acids grouped with cholesterol sulfate and the co-clustering of well-established OC biomarkers (CA125, HE4, MUC1). Together, these findings show that multi-omics integration provides an interconnected view of biological processes not visible within individual datasets, which supports a systems-level view of OC biology that can be exploited for diagnostic development as well as potential therapeutic interventions.

While this study provides the foundation for future multi-omics work in OC serum, it has several limitations. First, all lipidomic and metabolomic analyses included here are discovery-based, which relies on relative quantification and putative annotations. Untargeted analysis using annotation software such as Compound Discoverer can produce incorrect identifications, especially for low-abundance or structurally similar species. The findings outlined here therefore require verification using targeted, quantitative methods prior to their application in the development of a diagnostic tool. Furthermore, metabolites and lipids are often perceived as less stable biomarkers than proteins due to their rapid response to physiological and environmental inputs [61]. However, this dynamic behavior reflects sensitivity to underlying biological signals. In the context of cancer, tumor-driven metabolic alterations represent persistent sources of systemic change, so disease-associated signatures are detectable despite short-term variability due to infection [62], hormone cycles [63], or diet [64]. This responsiveness may, in fact, enable earlier detection of disease-associated changes compared to more static biomarker classes, particularly when combined into a multi-omics approach. Finally, while our findings overlap with the previous literature, the individual features and above questions await validation in independent cohorts.

Future work will prioritize targeted LC-MS verification of lipids, gangliosides, and metabolites identified in this dataset. Validating these findings in independent cohorts and investigating potential confounding factors such as age, body mass index, medications, and menopausal status will be important to evaluate the clinical relevance of these features and harmonize clinical attribute data across health systems.

A major contribution of this work is the characterization of the lipidome, gangliosome, and metabolome within a clinically relevant symptomatic cohort. By measuring molecular alterations in OC alongside non-malignant comparison groups in the same dataset, this study provides a comprehensive view of molecular remodeling within the broader diagnostic landscape. This work also highlights glycosphingolipid remodeling as an informative but understudied aspect of OC biology. Ultimately, this multi-omics resource is intended to serve as a foundation for hypothesis-driven follow-up studies aimed at developing biologically informed, multi-class biomarker strategies for early OC detection. 

## Figures and Tables

**Table 1:** Cohort sample distribution. Summary table of ovarian cancer cases, borderline tumors, and non-OC controls included in this symptomatic cohort study.

Diagnosis	Number of Samples	Percentage of Cohort
All ovarian cancer	185	36.8%
*Early-stage ovarian cancer*	*72*	*14.3*%
*Late-stage ovarian cancer*	*113*	*22.5*%
Borderline tumors	25	5.0%
Benign gynecological conditions	164	32.6%
GI disorders	49	9.7%
Healthy controls	80	15.9%
Total	503	100%

## Data Availability

The original contributions presented in this study are included in the article/Supplementary Material. Further inquiries can be directed to the corresponding author.

## Supplementary Materials

The following supporting information can be downloaded at https://www.mdpi.com/article/10.3390/diagnostics16142143/s1: Figure S1: Scatter plots of amino acids. Scatter plots depicting alterations across all 6 groups for individual amino acids. Healthy controls (healthy, dark blue), benign gynecological disorder (benign, medium blue), GI disorder (GI, light blue), borderline tumors (borderline, yellow), early OC (early-stage ovarian cancer, pink), late OC (late-stage ovarian cancer, red). Statistical significance is depicted by Kruskal-Wallis. Healthy controls (dark blue), benign (medium blue), GI disorder (light blue), borderline (yellow), early OC (early-stage ovarian cancer, pink), late OC (late-stage ovarian cancer, red). * *p* ≤ 0.05, ** *p* ≤ 0.01, *** *p* ≤ 0.001, **** *p* ≤ 0.0001; Table S1: Cohort Demographic Details. Detailed breakdown of relevant clinical details: age, race/ethnicity, histology, and stage.; Table S2: Cohort Disease States. Descriptions and examples of diagnoses included in the study design.

## Abbreviations

The following abbreviations are used in this manuscript:

ANOVA	Analysis of variance
ApoA1	Apolipoprotein A1
B2M	Beta-2 microglobulin
B3GALT4	Beta-1,3-galactosyltransferase 4
CA125	Cancer antigen 125
CD	Collisional dissociation
Cer	Ceramide
CPT1	Carnitine palmitoyl transferase 1
CV	Coefficient of variation
ELISA	Enzyme-linked immunosorbent assay
ESI	Electrospray ionization
FOLR1	Folate receptor α
GI	Gastrointestinal
HE4	Human epididymis protein 4
HGSOC	High-grade serous ovarian cancer
HMDB	Human Metabolome Database
IDO1	Indoleamine 2,3-dioxygenase 1
KEGG	Kyoto Encyclopedia of Genes and Genomes
LPC	Lysophosphatidylcholine
LPI	Lysophosphatidylinositol
*m*/*z*	Mass-to-charge ratio
MS	Mass spectrometry
MUC1	Mucin 1
OC	Ovarian cancer
PC	Phosphatidylcholine
PE	Phosphatidylethanolamine
PLS-DA	Partial least squares discriminant analysis
rcf	Relative centrifugal force
ROUT	Robust regression and outlier removal
RT	Room temperature
SM	Sphingomyelin
TCA	Tricarboxylic acid
TDO2	Tryptophan 2,3-dioxygenase
Tmix	Technical quality control mixture
UHPLC	Ultrahigh pressure liquid chromatography
VAS	Vague abdominal symptoms
