# Supplementary material for: Multi-Omics Profiling in a Symptomatic Cohort Identifies Coordinated Biomarker Signatures in Ovarian Cancer Serum
Source: Diagnostics (Basel). 2026 Jul 8;16(14):2143. doi: 10.3390/diagnostics16142143 (PMC13409400; doi:10.3390/diagnostics16142143)
Supplement: Supplementary file 1 [file diagnostics-16-02143-s001.zip › diagnostics-4269485-supplementary.pdf]

diagnostics

MDPI

Supplementary Materials:

Supplemental Table S1. Cohort Demographic Details. Detailed breakdown of relevant clinical details: age, race/ethnicity, histology, and stage.

	N = 503	
	OC<br>n = 185	Non-OC + Borderline<br>n = 318
<50	34	131
50-59	64	104
60-69	56	53
70+	30	23
Unknown	1	7
Mean (SD)	58.8 (11.4)	50.8 (13.4)
African American	0	1
Arab	0	0
Asian	0	0
Caucasian	69	117
Hispanic	24	3
Unreported	92	197
Serous	121	
Mucinous	16	
Endometrioid	15	
Clear Cell	12	
Mixed	4	
Non-Epithelial	1	
Not Reported	16	
Borderline		25
Benign		164
GI Disorder		49
Healthy controls		80
I	48	
II	24	
III	97	
IV	16	

Supplemental Table S2. Cohort Disease States. Descriptions and examples of diagnoses included in the study design.

Term	Description	Example
Serous	The most common subtype of epithelial OC that originates in the serous membrane covering fallopian tubes	Serous adenocarcinoma
Mucinous	Subtype of epithelial OC characterized by tumors containing mucus-producing cells	Mucinous ovarian carcinoma

Endometrioid	Subtype of epithelial OC. Histologically resembles endometrial tissue with features similar to endometrial cancer	Endometrioid adenocarcinoma
Clear Cell	Subtype of epithelial OC named for its cells' uniquely transparent appearance under a microscope	High-grade clear cell ovarian carcinoma
Mixed	Mixed epithelial OC arises when two or more distinct cell types from different histological subtypes of OC are present	Endometrioid ovarian carcinoma + mucinous differentiation
Non-Epithelial	Rare, diverse OC subtypes, do not originate from epithelial cells. Include germ cell tumors and sex-cord stromal tumors	Granulosa cell tumor
Not Reported	In some cases, OC pathology reports describing histological findings were not available	Unknown histology
Benign: mass	Non-cancerous tissue growth. Generally slow growing, do not invade nearby tissues, do not metastasize	Leiomyoma, fibroma, or cystadenofibroma
Benign: cyst	Sac-like pocket of membranous tissue. Contains fluid, air, or other substances	Dermoid cyst, serous cystadenoma, or endometrioma
Benign: growth	Abnormal increase in tissue size. Does not invade nearby tissue or metastasize, but may require monitoring or removal	Typical hyperplasia or fibrothecoma
Benign: other	Symptomatic but non-complex benign diagnoses with unclear or unavailable pathology	Unknown histology
GI Disorder	Gastrointestinal (GI) disorders present with a variety of symptoms, including abdominal discomfort	Diverticulitis or active chronic gastritis
Healthy controls	Otherwise healthy without active clinical diagnosis	No active clinical diagnosis

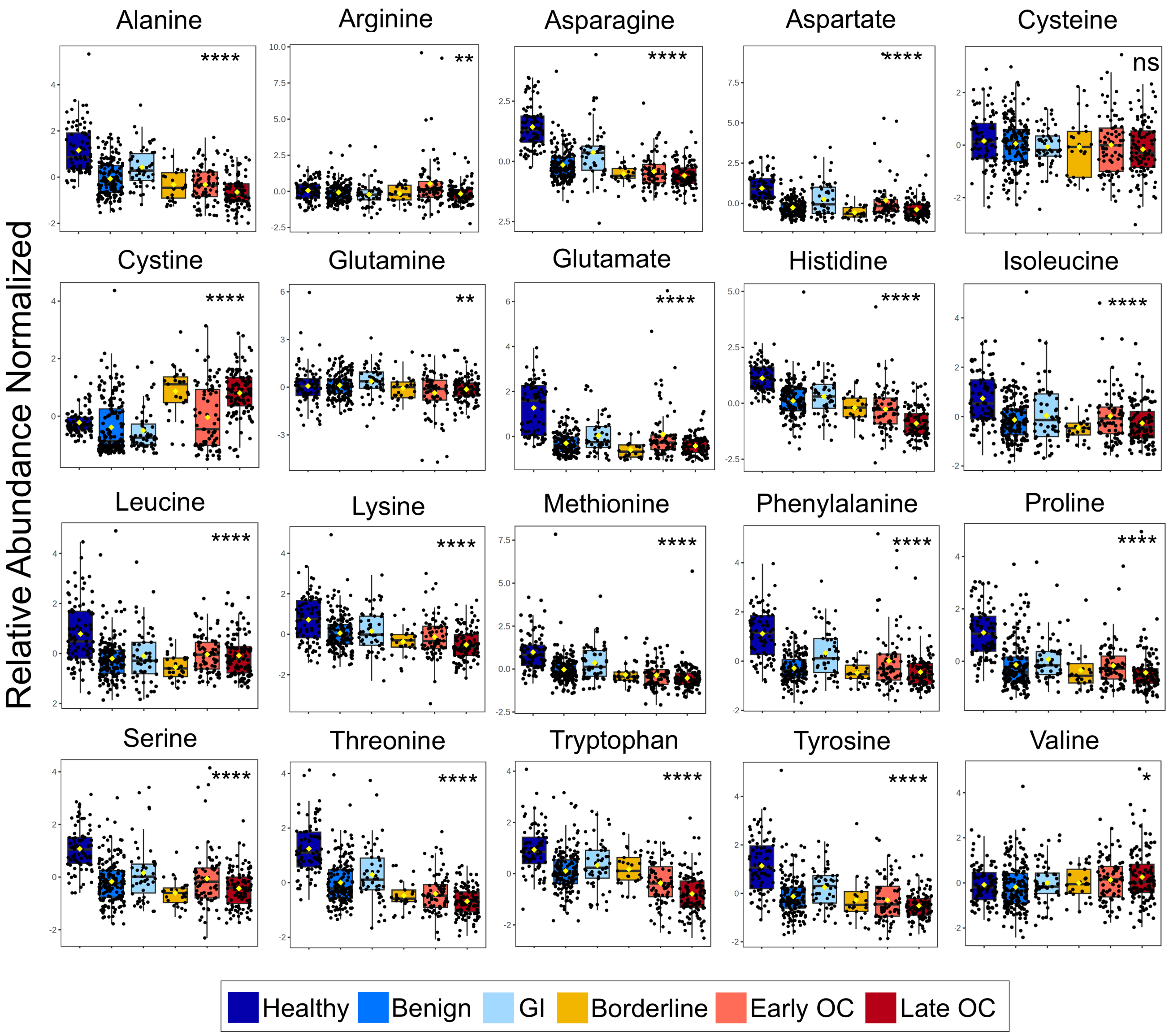

Supplemental Figure S1. Scatter plots of amino acids. Scatter plots depicting alterations across all 6 groups for individual amino acids. Healthy controls (healthy, dark blue), benign gynecological disorder (benign, medium blue), GI disorder (GI, light blue), borderline tumors (borderline, yellow), early OC (early-stage ovarian cancer, pink), late OC (late-stage ovarian cancer, red). Statistical significance is depicted by Kruskal-Wallis. Healthy controls (dark blue), benign (medium blue), GI disorder (light blue), borderline (yellow), early OC (early-stage ovarian cancer, pink), late OC (late-stage ovarian cancer, red). * $p \leq 0.05$, ** $p \leq 0.01$, *** $p \leq 0.001$, **** $p \leq 0.0001$
